# Supplementary material for: A pH Dual-Responsive Multifunctional Nanoparticle Based on Mesoporous Silica with Metal-Polymethacrylic Acid Gatekeeper for Improving Plant Protection and Nutrition
Source: Nanomaterials (Basel). 2022 Feb 18;12(4):687. doi: 10.3390/nano12040687 (PMC8875777; doi:10.3390/nano12040687)
Supplement: Supplementary file 1 [file nanomaterials-12-00687-s001.zip › nanomaterials-1556667-supplementary.pdf]

Legends

Figure S1. 3D structure simulation diagram of Prochloraz molecule by ChemBio3D Ultra 12.0.

Figure S2. Bioactivities of the of BMMs and BMMs-PMAA/Fe3+ against *Rhizoctonia solani.*

Figure S3. SEM (A, B) and EDS (C,D) mapping characterizations of the rice plant root treated with deionized water and BMMs-PMAA/Fe^{3+} nanoparticles.

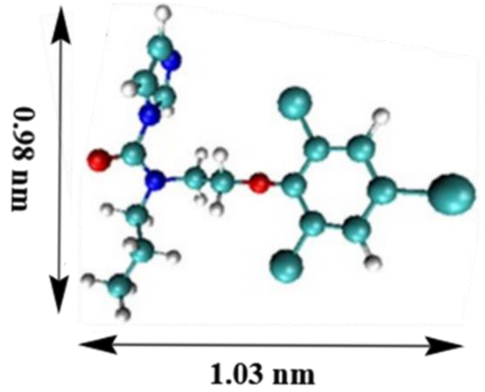

Figure S1. 3D structure simulation diagram of Prochloraz molecule by ChemBio3D Ultra 12.0 (PerkinElmer, Waltham, MA, USA).

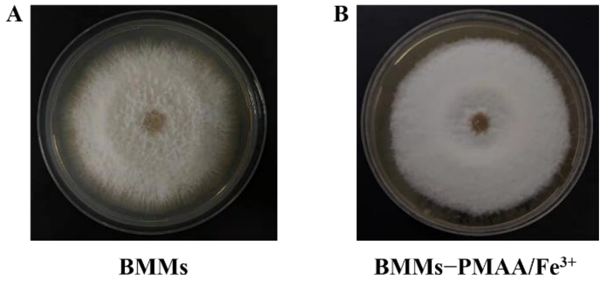

Figure S2. BMMs−Bioactivities of BMMs (**A**) and BMMs−PMAA/Fe^{3+} (**B**) against *Rhizoctonia solani.*

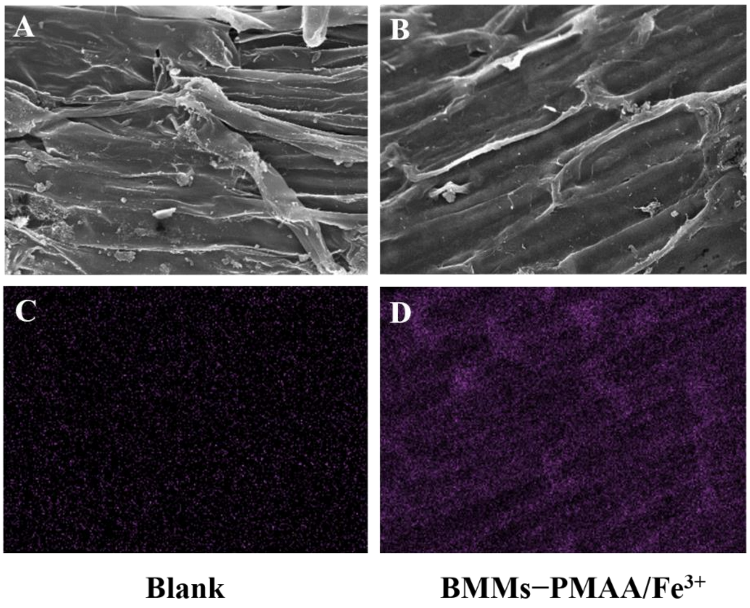

Figure S3. SEM images of the rice plant root treated with deionized water (**A**) and BMMs−PMAA/Fe^{3+} nanoparticles (**B**), EDS mapping characterizations of the rice plant root treated with deionized water (**C**) and BMMs−PMAA/Fe^{3+} nanoparticles (**D**).
